# Immune responses to dengue virus in the skin

**DOI:** 10.1098/rsob.180087

**Published:** 2018-08-22

**Authors:** Abhay P. S. Rathore, Ashley L. St. John

**Affiliations:** 1 Program in Emerging Infectious Diseases, Duke-National University of Singapore, Republic of Singapore; 2 Department of Pathology, Duke University Medical Center, Durham, NC, USA; 3 Department of Microbiology and Immunology, Yong Loo Lin School of Medicine, National University of Singapore, Republic of Singapore

**Keywords:** dengue virus, skin, mast cells, dendritic cells, pattern recognition receptors, mosquito saliva

## Abstract

Dengue virus (DENV) causes infection in humans and current estimates place 40% of the world population at risk for contracting disease. There are four DENV serotypes that induce a febrile illness, which can develop into a severe and life-threatening disease in some cases, characterized primarily by vascular dysregulation. As a mosquito-borne infection, the skin is the initial site of DENV inoculation and also where primary host immune responses are initiated. This review discusses the early immune response to DENV in the skin by both infection target cells such as dendritic cells and by immune sentinels such as mast cells. We provide an overview of the mechanisms of immune sensing and functional immune responses that have been shown to aid clearance of DENV *in vivo*. Finally, we discuss factors that can influence the immune response to DENV in the skin, such as mosquito saliva, which is co-injected with virus during natural route infection, and pre-existing immunity to other DENV serotypes or to related flaviviruses.

## 1. Dengue virus infection and clinical presentation

Dengue virus (DENV) is a vector-borne human pathogen that belongs to the family of *Flaviviridae*. The disease caused by DENV is considered to be one of the most important human viral maladies of the twenty-first century with nearly 390 million infections annually worldwide in recent years [1]. Epidemiological estimates considering the tropical and sub-tropical distribution of the virus suggest that as many as 3.9 billion people are at risk of contracting the disease [2]. DENV is primarily transmitted to humans by a bite from the urban mosquito, *Aedes aegypti*, which deposits virus particles in the skin while probing for a blood meal [3], making the skin the initial site of immune defence against it. DENV has four antigenically distinct serotypes, DENV 1–4, which adds complexity to this disease since humans often experience more than one DENV infection in a lifetime. Specific immunity against a homologous serotype is long-lasting and protective. However, humans can be infected with a new serotype multiple times since immunity is cross-reactive but non-neutralizing for a heterologous strain [4]. Several other important human pathogens that are closely related to DENV within the genus *Flavivirus* include West Nile virus (WNV), Japanese encephalitis virus (JEV), yellow fever virus (YFV) and the newly-emerged Zika virus (ZIKV).

DENV infection in humans causes a febrile illness known as dengue fever (DF). After injection of DENV virus particles into the skin, there is an incubation period of 4–7 days before symptoms arise, after which they can last for approximately 5 days [5]. Increasingly, we understand that some individuals experience asymptomatic or sub-clinical disease [1]. For those with clinically apparent DF, the signs and symptoms include headache, nausea, retro-orbital pain, muscle and joint pain, fever and rash. Haemorrhagic manifestations, such as petechiae, also occur in some DF cases. A reduction in platelet counts, or thrombocytopenia, is one of the hallmarks of DENV infection and this, like most other signs of disease, is usually self-resolving [6]. However, in some cases, DENV infection can progress to life-threatening severe complications such as dengue haemorrhagic fever (DHF) and dengue shock syndrome (DSS). Severe disease is characterized by a dramatic or rapid decline in platelet counts, and severe bleeding, multi-organ involvement and hypovolaemic shock can occur in some cases. In its most severe form, DENV infection is life-threatening.

Currently, there are no approved therapeutics for the treatment of DENV infection or for the targeted prevention of severe disease, although multiple vaccine candidates are currently at various stages of clinical development and testing. For some other viral infections, it has been shown that delivery of antigens or vaccine formulations into the skin has comparative advantages over other routes of administration [7], demonstrating the uniqueness of this site of inoculation. Therefore, understanding the initial events of immune responses to DENV infection in the skin that occur during natural route infections is crucial for our progress towards robust intervention strategies against DENV, including therapeutics and vaccinations.

## 2. Initiation of natural route infection through the skin

DENV is injected into the skin by the female mosquito whose mouth parts, or proboscis, probe through the epidermis into the dermis until a suitable site is found where blood can be collected from a capillary. During each probing event, saliva containing virus particles may be injected [8,9]. Estimates from studies that examined feeding of DENV- or WNV-infected mosquitoes on mice have suggested that approximately 1 × 10^4^ to 1 × 10^6^ PFU of viruses are injected by mosquitoes into a single site while probing [10,11], while the volume of mosquito saliva injected is less than 1 µl (between 1 and 5 nl were measured in a recent quantification of forced salivation) [12]. It is assumed that viruses are primarily injected into the dermis; however, studies of the deposition of malaria parasite *Plasmodium berghei* showed that sporozoites were also detected in the epidermis [13], which is also likely to occur for arboviral pathogens since the deposition of saliva and probing behaviour is similar. This is relevant for understanding which immune and infection target cell types may encounter the virus upon natural route infection. Although the inoculum of DENV is relatively small, it is able to efficiently establish infection in the host.

After skin infection, DENV must achieve systemic infection in order to complete its transmission cycle by infecting new mosquito hosts [14]. DENV is a lymphotropic pathogen and uses the lymphatics and lymph nodes as a conduit to the blood. The lymphotropic nature of DENV was first shown in primates that were inoculated into the skin. Infection became detectable sequentially in draining lymph nodes prior to reaching systemic infection where virus could be detected in the serum [15]. Interestingly, even after viraemia subsided, DENV remained detectable in the site of inoculation, indicating a persistence of infection in the skin [15]. In contrast to the site of initial virus infection, areas of skin with pathologic manifestations during the acute systemic stage of disease, which may be characterized by haemorrhaging or oedema, are usually not positive for DENV infection and, thus, these lesions are likely to be the result of the systemic inflammatory response [16]. Surprisingly, in one study, the early presence of rash or cutaneous manifestations of disease was associated with improved disease outcomes in human patients [17]. However, other studies noted that immune cells in the skin were activated at the onset of DSS [18], potentially reflecting the systemic inflammatory response.

## 3. Infection target cell types in the skin and virus entry receptors

At the site of inoculation in the skin, key targets of DENV infection are immune cells of the myeloid lineage that are phagocytes, including various subsets of dendritic cells (DCs) and monocytes. In interferon (IFN)-α/β receptor knockout (*Ifnar*^–/–^) mice, CD103^+^ DCs, Ly6C^–^ CD11b^+^ DCs, and macrophages were all initial targets of infection, after which the monocyte-derived DCs (Ly6C^+^ CD11b^+^) became the primary infection targets [19]. Monocyte-derived DCs are significantly recruited into the skin following DENV infection in mice [19]. In human skin explants and sites of DENV vaccination, Langerhans cells were also targets of infection [20,21], as well as CD1c^+^ and CD14^+^ dermal DCs [21]. Interestingly CD141^+^ DCs, which make up a minority population in the skin, were not observed to be infected [21]. Macrophages and CD1c^+^ and CD14^+^ dermal DCs that are matured with IL-4 have also been shown to be more susceptible to DENV infection, potentially due to the upregulation of DENV receptors, DC-specific ICAM3-grabbing non-integrin (DC-SIGN) and the mannose receptor, in response to the cytokine treatment [22]. In spite of being infection targets, monocyte/macrophage lineage cells could play a dual role in infection since it has been shown that depletion of macrophages leads to early reduced infection in lymph nodes, yet to later increased infection burden *in vivo* systemically [23]. That study, however, examined the role of macrophages after systemic inoculation in mice lacking IFN-α/β/γ receptors, rather than specifically examining their function after skin inoculation [23], so the functional contributions of the skin-resident macrophage monocytes to infection amplification versus clearance are not yet characterized.

In the skin, multiple non-haematopoietic cell types are also thought to be early infection targets. *In vitro* or *ex vivo*, human fibroblasts have been shown to be infected, but this may be DENV strain-dependent [24,25]. Keratinocytes were also postulated to be DENV infection targets based on staining of cells morphologically consistent with keratinocytes for non-structural antigens in human skin explants after *ex vivo* infection [26]. A recent study has suggested, also using skin explants, that as much as 60% of the total infected cells in the skin may be keratinocytes [27]. However the proportion of keratinocytes infected was lower in another similar study [21], which may be attributable to differing methods or virus strains. Explants also lack the potential of exhibiting normal recruitment of cell types from the circulation, such as monocytes, which are highly permissive to DENV infection in animal models [19].

DENV infects the cell through receptor-mediated endocytosis and various receptors have been identified or proposed for DENV entry. Proteoglycan, heparin sulphate and glycosaminoglycans, which are commonly expressed on various mammalian cell types, have been shown to bind DENV *in vitro* [28]. Others have identified carbohydrate moieties on glycosphingolipids to be involved in DENV attachment to the cell surface [29,30]. Importantly a C-type lectin receptor, DC-SIGN molecule, was identified as an attachment receptor for DENV in DCs [31]. Similarly, the mannose receptor expressed on macrophages was shown to bind to DENV for its internalization. Blocking the mannose receptor using a specific antibody inhibited DENV infection of primary human macrophages [32]. More recently, phosphatidylserine receptors, TIM and TAM, were identified as attachment receptors for DENV entry in human primary kidney epithelial cells as well as primary astrocytes [33]. Other less defined co-receptors for DENV include CD14, HSP70, HSP90, GRP78 and claudin-1 [34–37]. Receptors for certain cells in the skin, such as keratinocytes, which are exposed to DENV during the cutaneous infectious mosquito bite, remain unknown.

## 4. Cellular sentinels to dengue virus infection

**Figure 1.**
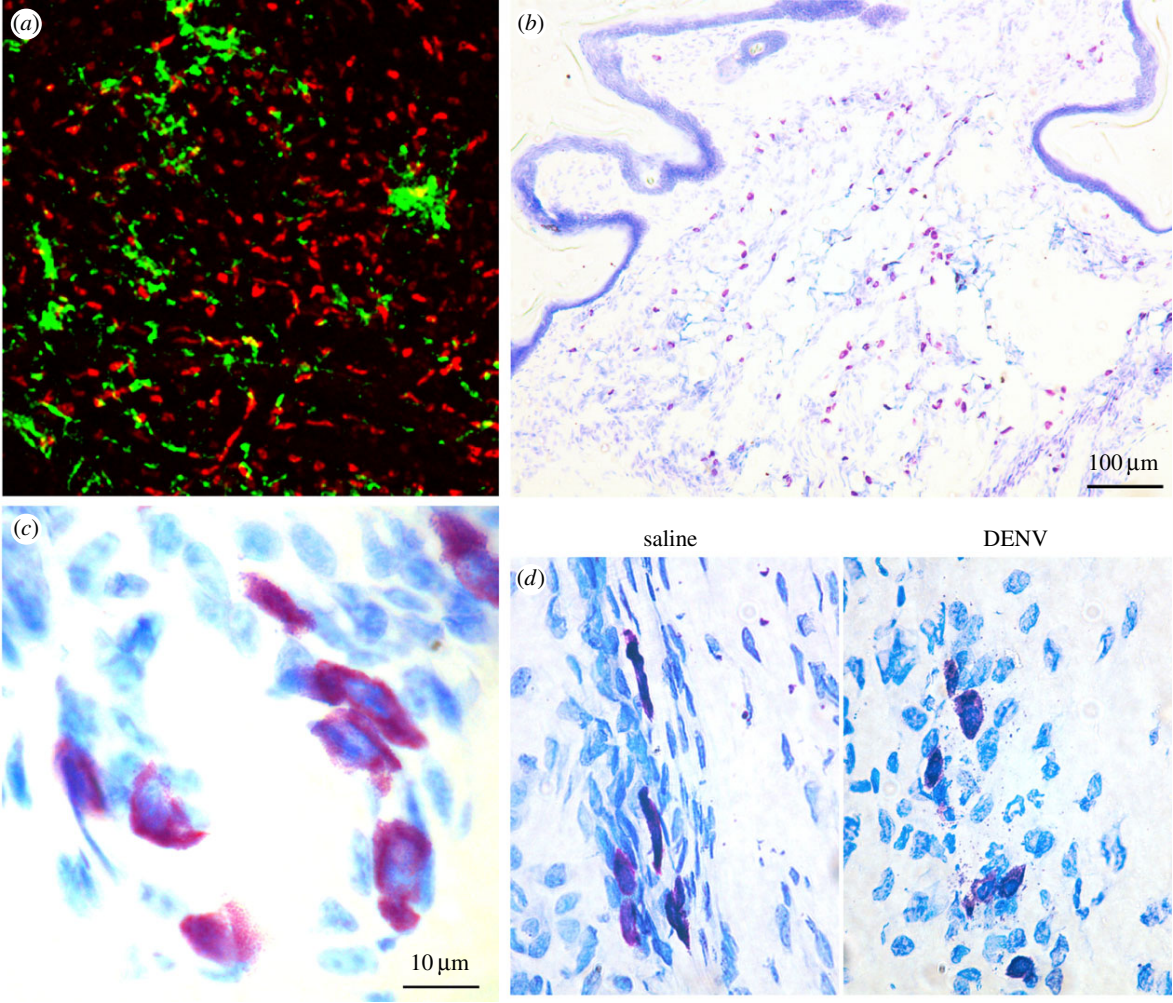
Immune cells are densely populated in the skin and are sentinels for DENV. (*a*) Whole mount mouse (C57BL/6) ear tissue stained for CD11c^+^ dermal DCs (green) and MC heparin (avidin, red). (*b*) Toluidine blue staining of a tissue section of uninfected mouse footpad shows MCs (deep purple) throughout the dermis. (*c*) A higher magnification image shows MCs densely packed with granules within the footpad skin. (*d*) MC degranulation in the skin visualized by toluidine blue staining of DENV-infected or saline control-injected tissue sections, 6 h following injection.

At the time of virus inoculation into the skin, DENV is not unopposed by the host but, rather, detected quickly by immune sentinels. Skin-resident immune cells of haematopoietic lineage that DENV encounters include Langerhans cells, DCs, macrophages and mast cells (MCs). In the healthy human dermis, MCs, DCs and macrophages are found at approximately similar densities: approximately 70–100 MCs, approximately 60 DCs (defined as CD11c^+^) and approximately 80 macrophages (defined as CD163^+^ FXIIIA^+^) per mm^2^ in tissue sections [38–40]. Images of MCs and DCs in the skin are provided in figure 1. MCs, which are granulated cells (figure 1*b*,*c*), are sentinels for DENV infection. They are distributed at relatively even intervals in the dermis but at highest concentration at the epidermal–dermal junction [38,39]. They also adopt a perivascular formation around blood and lymphatic vessels [41]. MCs are preloaded with granules (figure 1*c*) containing immune mediators and can respond to pathogens within minutes by releasing their granular contents into the extracellular environment (figure 1*d*). They can also produce inflammatory mediators *de novo*, either through enzymatic activation (e.g. production of eicosanoids) or transcriptional activation (e.g. cytokines, such as TNF). Often, the transcriptional activation programmes are pathogen-specific [42] and for DENV, MCs also induce pro-inflammatory transcriptional programmes *in vivo* [43]. DENV, as well as inactivated DENV particles, induces a degranulation response by MCs that promotes oedema and recruitment of cytotoxic cells [44,45]. MC-derived TNF that is associated with granules has been shown to initiate lymph node hypertrophic responses which are critical for timely induction of adaptive immunity [41,46], and MC-derived TNF in the skin leads to upregulation of E-selectin on vascular endothelium, which facilitates homing of immune cells into the tissue [47]. Functional studies in immune-competent mice showed that MCs contribute significantly to DENV clearance since mice deficient in MCs have augmented infection in draining lymph nodes [44]. Thus MCs are key early sentinels for DENV infection in the skin and have the ability to regulate skin inflammatory and lymph node responses.

Although degranulation of MCs does not require virus replication, mast cells are also able to internalize DENV, which produces replication intermediates such as dsRNA, and triggering of cytosolic pattern recognition receptors [44,48]. Internalization and activation receptors specific for DENV on MCs are not yet known. However, infection of cultured MCs with a mature morphology produced very little infectious virus, with less than 3% of the cultured cells infected based on infectious virus quantification by plaque assay [44]. Recently, using MCs isolated from human skin explants, it was suggested that a higher proportion of MCs could be infected with DENV based on staining for the structural component, capsid antigen [49]. However, further validation of productive replication is needed, such as confirming production of replication intermediates and infectious virus particles, to conclusively establish that MCs are a DENV replication target in human skin. If MCs can sustain virus replication, it may be at low levels since it has been shown that more than 90% of the haematopoietic lineage cell types infected in the human skin are various subsets of DCs [21]. Thus, the role of MCs in DENV infection appears to be more in line with one of early host defence rather than virus amplification.

**Figure 2.**
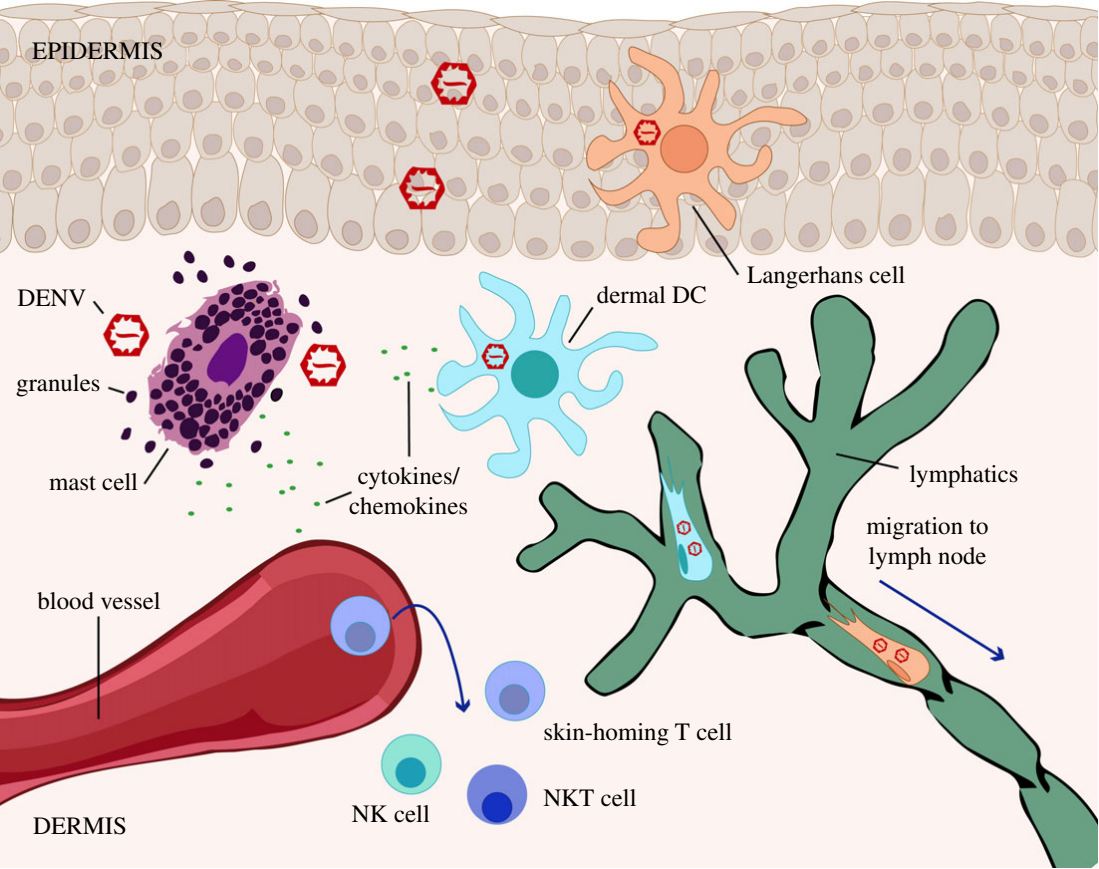
A schematic of immune responses initiated in the skin upon DENV infection. Diagram shows the network of immune cell types that encounter DENV in the skin in the early hours following infection. Limited virus is thought to be deposited in the epidermis during natural route infection, but Langerhans cells in that location are infection targets. In the dermis, DCs are also prime infection targets. MCs, which are not substantially infected in the skin, are activated by DENV and degranulate. Their activation leads to the recruitment of NK and NKT cells to the site of infection. Skin-homing T cells are also thought to be recruited into skin sites of infection. Using lymphatics, infected DCs carry DENV to the draining lymph node.

Notably, many of the cell types that are early infection targets in the skin, including dermal DCs, Langerhans cells and macrophages, are also antigen presenting cells, which may contribute to the development of adaptive immune responses in spite of being confirmed infection targets. The two subsets of dermal DCs that were observed to be infected in the skin, CD1c^+^ and CD14^+^ DCs, have responsibilities for migrating to draining lymph nodes to induce systemic T cell and T follicular helper cell responses [21]. That DENV antigen can be found in each of these subsets suggests that DENV antigen is likely to be presented for these purposes, although the differential contributions of these subsets to infection amplification, immunity or infection outcomes have not been described. A diagram summarizing cellular trafficking and activation in the skin following DENV infection is provided as figure 2.

## 5. Innate immune pathways important for dengue virus detection and protection

Even cellular targets of DENV infection have important immune pathways to resist virus infection. For example, there are multiple pathogen recognition receptors (PRRs) that can recognize DENV replication intermediate products. These innate immune pathways are evolutionarily conserved and may evoke similar responses in multiple tissues, although the skin would be unique in that it is the first site of exposure to DENV and the location of the first infection target cell populations. Once DENV is internalized within cells, dsRNA intermediates are formed during the replication of the viral genome [50]. Importantly, this critical stage of the DENV replication cycle can be sensed by PRRs expressed by various susceptible cell types in the skin [51]. Membrane anchored toll like receptors (TLRs) such as TLR-3 and TLR-7 and cytoplasmic retinoic acid inducible gene I (RIG-I) like receptors, such as RIG-I and MDA-5, can recognize single or double-stranded complementary viral RNA molecules, which initiates a cascade of signalling events crucial for antiviral defence [52,53]. TLR-3 activation leads to the activation of interferon regulatory transcriptional factors IRF-3, IRF-7 and as well as NF-κB. This results in transcriptional upregulation of Type I (α and β) interferons, various cytokines and other interferon-stimulated genes [54,55]. *In vitro* studies, some using relevant skin cell types such as keratinocytes, have demonstrated that DENV replication is detected by TLR-3 and that this contributes to production of Type I interferons and the cytokine IL-8 [56–58]. Similar to TLR-3, MDA-5 and RIG-I are also known to sense dsRNA intermediates during DENV replication [58,59]. In general, triggering of RIG-I and/or MDA-5 leads to activation of IRF-3, which together with TBK-1 and NF-κB stimulates the production of IFN-β and other IFN-stimulated genes [53]. Studies using a RIG-I agonist have shown that activation of RIG-I boosts the host cell's innate antiviral response, which limits DENV replication [60]. Furthermore, these molecules regulate the production of chemokines that recruit cytotoxic NK1.1^+^ T cells, for example CXCL10. CXCL10 production by sentinel MCs was shown to be dependent on RIG-I and MDA5 [44]. IFNs and IFN-response genes are essential for the initial containment of DENV at the cutaneous infection site before an adaptive immune response is established. More recently, recognition of DENV replication was also shown to be mediated by cytoplasmic DNA sensor cyclic GMP-AMP synthetase (cGAS) by various direct and indirect mechanisms [61,62]. Most innate immune receptors relevant for DENV detection are adapted to detect viral nucleic acid or replication intermediates; therefore, it should also be noted that some of these products could be produced or present in the case of abortive infection [63], so successful completion of the virus replication cycle is not necessarily required for cellular activation to DENV and it may occur in cell types that are not traditional infection targets.

IFN production is a key goal of PRR activation for viral pathogens and DENV is highly susceptible to effective induction of both Type I and Type II interferons. This is supported by the increased susceptibility of IFN-receptor deficient mouse models to DENV infection. Furthermore, it has been shown that DENV more efficiently antagonizes the IFN pathway in humans compared with mice due to differential binding of DENV NS5 protein with mouse and human STAT2 [64]. Although antagonized, human cells still induce high levels of IFN production in response to DENV, so this pathway is not entirely abrogated in humans during infection [65,66]. Several varied mechanisms that DENV uses to resist innate host defence by antagonizing PRR and IFN pathway signalling have been recently reviewed [67]. However, when induced, IFN plays an important role in varied innate and adaptive immune processes including resistance to viral entry, promotion of T follicular helper cell activity and effective antibody class-switching responses [68–70]. IFN production by DCs that are infected and/or presenting DENV antigen and uninfected bystander cells activated by the inflammatory environment would likely influence these processes.

## 6. Dengue virus clearance by NK cells and skin homing T cells

Recruitment of cells that contribute to DENV clearance is another important step in the containment of virus infection and initiation of adaptive immune responses, which occurs subsequent to the initial sentinel response of skin-resident immune cells. As discussed above, cytotoxic cells such as NK cells, NKT cells and CD8^+^ T cells are recruited into the DENV-infected skin in a MC-dependent manner [43]. NK cells are able to kill DENV-infected DCs via both antibody-dependent and independent mechanisms [71,72]. In mice, depletion of NK1.1^+^ cells leads to greatly enhanced titres of virus in the draining lymph nodes by 24 h post-infection, indicating the important contributions of NK and/or NKT cells to infection clearance in peripheral tissues at early time points [44]. Consistent with this, higher numbers of activated NK cells in the blood of human DENV patients have been associated with milder disease [73]. Interestingly, during acute dengue infection in humans, activated CD8 T cells in the blood have a skin homing phenotype, involving expression of CXCR3, CCR5 and the skin-homing marker cutaneous lymphocyte-associated antigen (CLA) [74]. Together, these data indicate that skin-homing of cytotoxic cells is an important component of DENV immunity and clearance.

## 7. Mosquito and host factors that can influence infection outcomes

The natural route of infection for DENV is the mosquito bite, which likely influences the infectivity of DENV and inflammation elicited by it through both the components of the mosquito saliva and the process of probing for a blood meal involving physical damage to the tissue. However, little is known about the influence of the mosquito bite on DENV and the results from multiple studies are potentially contradictory. On the one hand, mosquito bites are immunostimulatory. For example, it has been shown that MCs are strongly activated by a mosquito bite so that they degranulate within the skin. The mosquito bite-induced ‘wheal and flare’ reaction is attributed to MC histamine [75]. Yet, in contrast, mosquito saliva has also been described as having some immunosuppressive properties [76]. The few recent reports examining innate responses to viruses delivered by mosquito bite have suggested that mosquito saliva may enhance the infectivity of those viruses [77,78]. Interestingly, since it has been shown that IL-4 can potentiate infectivity, it is possible that the mosquito bite could be enhancing for infection through its induction of IL-4 [78–80]. Mosquito saliva may also influence the cellularity in the skin. One study examining the influence of the mosquito bite on the infectivity of another arbovirus, Semliki Forest virus, showed that the mosquito bite induces influx of neutrophils which hinder viral clearance at the site of infection [81]. A recent study also showed immune cell (monocytes, macrophages and DCs) migration in the skin was increased in the presence of mosquito salivary gland extracts, as well as in the presence of DENV enhancing antibodies, leading to exacerbated DENV disease in IFN Type-I deficient mice [82]. *Aedes aegypti* saliva has also been shown to suppress transcription of key virus detection genes at the site of infection, such as TLR-7, RelA, IFN-γ and IP-10, which was postulated to hinder the detection of the initial virus inoculum and contribute to increased viral titres *in vivo* [83]. In contrast, experiments using human donor-derived DCs suggested a protective role for mosquito saliva and showed inhibition of DC infection by DENV in the presence of saliva [84]. Mosquito saliva is a complex mixture of numerous proteins, which require further studies to understand and describe their functions *in vivo* in terms of modulating arboviral infections.

Inoculation of virus into the skin may also occur into a host that has pre-existing immunity, whether to a homologous serotype, a heterologous serotype or to a *Flavivirus* from a related serocomplex. Although concentrations of antibodies are very low in the interstitial space [85], they are likely to be present at higher concentrations as a result of mosquito piercing of blood vessels or due to oedema and vascular permeability at the site of infection. There are multiple ways that antibodies could influence infection, whether through our traditional understanding of antibody-dependent enhancement of infection or via alternative pathways. For example, in the case of DENV, antibodies to a heterologous DENV serotype could enhance infection of Fc-receptor bearing cells, monocytes and macrophages, known as antibody-dependent enhancement of infection (ADE), resulting in severe disease [86–88]. However, antibodies that are cross-reactive to other closely related viruses could also potentiate immunity, as was shown to occur due to the presence of serocomplex-cross reactive antibodies after vaccination. In that case, antibodies may bind to the virus particles, promote their uptake and increase the presentation of the viral antigens in draining lymph nodes [89]. DENV-immune complexes have been shown to enhance infection of Fc-receptor bearing cells including DCs [90] and MCs [91], but they also can trigger activation and enhanced degranulation of MCs compared to exposure of MCs to virus alone (without cross-reactive antibodies) [92]. This IgG-enhanced degranulation to DENV has been shown to occur via cross-linking of the FcγRIII receptor in mice [92]. Furthermore, antibodies can promote antibody-dependent cell-mediated cytotoxicity (ADCC) of infected cells by NK cells, which has the potential to enhance killing of infected cells [71]. Of course, the specificity and quality of antibodies would greatly influence the potential outcomes. As we know, both concentration dependent and specificity dependent changes can influence whether antibodies result in ADE or promote neutralization [87].

Finally, T cells, which are under-represented in the literature with respect to DENV infection of the skin, can also have immune memory functions. Some studies have observed that cross-reactive T cells are detrimental to recovery from DENV due to the phenomenon of original antigenic sin [93], yet other recent studies have indicated that serocomplex cross-reactive T cell responses can promote protection during a heterologous challenge [94,95]. Further studies are needed to understand how memory T cells influence a homologous or heterologous DENV infection in the skin.

## 8. Closing remarks

DENV infection begins in the skin where the immune response is composed of multiple immune cell types that are also potentially targets of infection and enhanced cellular trafficking to and from the site of infection (figure 2). The magnitude and character of the initial immune response can influence the viral burden at later time points and the kinetics of virus clearance. This natural route of infection is also representative of the route used for vaccine administration, making it important to understand the initial responses that are immunogenic and protective, as well as which host factors have the potential of modulating anti-DENV immunity in the skin.

## Data accessibility

This article has no additional data.
